# Prevalence of blood type A and risk of vascular complications following transcatheter aortic valve implantation

**DOI:** 10.1007/s12471-016-0804-z

**Published:** 2016-02-09

**Authors:** M.-T. Rofe, Y. Shacham, A. Steinvi, L. Barak, M. Hareuveni, S. Banai, G. Keren, A. Finkelstein, H. Shmilovich

**Affiliations:** Department of Cardiology, Tel Aviv Sourasky Medical Center, Affiliated to the Sackler Faculty of Medicine, Tel Aviv, Israel; Department of Haematology, Tel Aviv Sourasky Medical Center, Tel Aviv, Israel

**Keywords:** Transcatheter aortic valve implantation, Aortic stenosis, Blood type A, Vascular complications

## Abstract

**Objectives:**

To assess the prevalence of blood type A among patients referred for transcatheter aortic valve implantation (TAVI) and whether it is related to vascular complications.

**Backgrounds:**

Vascular complications following TAVI are associated with adverse outcomes. Various blood types, particularly type A, have been shown to be more prevalent in cardiovascular diseases and to be related to prognosis.

**Methods:**

The prevalence of various blood types in a cohort of 491 consecutive patients who underwent TAVI was compared with a control group of 6500 consecutive hospitalised patients. The prevalence and predictors of vascular complications and bleeding events were evaluated in the blood type A group and were compared with non-type A patients.

**Results:**

The mean age of TAVI patients was 83 ± 6 years, and 40 % were males. Patients were divided into two groups: blood type A (*n* = 220) and non-type A (*n* = 271). Type A was significantly more prevalent in the TAVI group than in the control group (45 vs. 38 %, *p* = 0.023). Compared with the non-type A group, patients with blood type A had more major and fatal bleeding (14.5 vs. 8.1 %, *p* = 0.027) and more vascular complications (any vascular complication: 24.5 vs. 15.9 % *p* = 0.016; major vascular complications: 12.3 vs. 7 % *p* = 0.047). In a multivariable analysis, blood type A emerged as a significant and independent predictor for vascular complications and bleeding events.

**Conclusions:**

Blood type A is significantly more prevalent in TAVI patients than in the general population and is related to higher rates of vascular and bleeding complications.

## Introduction

Severe aortic stenosis is a major cause of morbidity and mortality in the elderly [1]. The risk of surgical aortic valve replacement rises dramatically in relation to comorbidities which can be evaluated using the Euroscore system [2, 3] thus deferring a large group of patients from surgery. In the last decade, transcatheter aortic valve implantation (TAVI) was shown to confer a lower risk of morbidity and mortality in this subset of patients with a high surgical risk and has now become common practice [4–6], but vascular complications after TAVI are associated with adverse short- and long-term outcomes [6–8].

Recent studies have confirmed that the ABO locus that encodes for the ABO blood type may be associated with myocardial infarction [9] and venous thromboembolism [10, 11]. Non-O blood types, mostly type A, were related to increased prevalence of morbidity and mortality in various cardiovascular diseases [12–14] but this was not studied in patients undergoing TAVI.

We investigated the prevalence of blood type A in TAVI patients vs. its prevalence in the general population and whether it is related to periprocedural vascular complications as compared with non-type A patients who underwent TAVI.

## Methods

### Study population

The data for the present study were collected between the years 2009 and 2014 at a single tertiary-care facility [15]. The study was approved by the institutional ethics committee and written informed consent was obtained from each patient. Patients were recruited during their participation in the Tel-Aviv Angiography Prospective Study [16]. The diagnosis of severe symptomatic aortic stenosis was based on clinical, echocardiographic and haemodynamic criteria [17]. Suitability and eligibility for TAVI were determined by a joint team consisting of an interventional cardiologist, a cardiac surgeon, and an echocardiographer. The control group representing the general population consisted of 6500 consecutive patients who were hospitalised and underwent blood type sampling for other clinical reasons.

### TAVI procedure

Two types of aortic valve prostheses were implanted: The CoreValve prosthesis (Medtronic, Minneapolis, MN, USA) and the Edwards Sapien or Sapien XT prosthesis (Edwards Lifesciences, Irvine, California). For all TAVI procedures, three senior interventional cardiologists performed the peripheral aspects of the TAVI procedures (introduction of the sheaths through the femoral artery, Prostar closure device deployment, and the suturing of the entry ports). Valve type and size were planned prior to the procedure according to preprocedural clinical, echocardiographic, angiographic and CT parameters and at the discretion of the senior interventional cardiologist. The available valve sizes for the Edwards Sapien XT prosthesis were 23 and 26 mm and the valve sizes for the CoreValve prosthesis were 26, 29, and 31 mm [15].

### Definition of vascular complications

The original consensus report of the Valve Academic Research Consortium (VARC-1) standardised the endpoint definitions of TAVI procedures including the occurrence of vascular complications [18]. During 2012, these endpoints were updated – known as VARC-2 [19]. While the general definitions of vascular complications were unchanged following the update, VARC-2 incorporated a more rigorous approach to bleeding and haemoglobin decline following TAVI, stating that major bleeding definitions (a haemoglobin drop > 3 g/dl or transfusion of two or more packed red blood cell units) should also be considered to be major vascular complications, while in the former VARC-1 endpoints, only transfusion of ≥ 4 packed red blood cell units was considered a major vascular complication. Importantly, in both consensus documents, interventional or surgical repair for failed percutaneous closure during the initial procedure without other clinical consequences was considered a minor vascular complication [18, 19]. Accordingly, we classified vascular complications using VARC-2.

### Statistical analysis

All data are displayed as mean (± standard deviation) for continuous variables, and as the number (percentage) of patients in each group for categorical variables. The Student’s t-test and *χ*^2^ test were used to evaluate the statistical significance of differences between continuous and categorical variables, respectively.

Logistic regression models used any vascular complications, or the combined outcome variable of any vascular complication or major bleeding, as the dependant variables, and they were adjusted to age, gender, body mass index (BMI), diabetes mellitus, hypertension, estimated glomerular filtration rate, previous stroke, previous cardiac surgery, Society of Thoracic Surgeons risk model (STS) score, ejection fraction and anaemia. Cox proportional hazard models for all-cause mortality were adjusted separately for major vascular complications as well as to other predictors of mortality following TAVI [5, 20]: gender, age, BMI, systolic heart failure, prior coronary artery bypass graft, prior percutaneous coronary intervention, atrial fibrillation, pulmonary disease, STS score, preoperative mean gradient, and creatinine clearance test. Analyses were considered significant at a two-tailed p value of less than 0.05. SPSS statistical package was used to perform statistical evaluation (SPSS, Chicago, IL).

## Results

The study population who underwent TAVI included 491 patients with a mean age of 83 ± 6 years (range 61–98), of whom 196 (40 %) were males. When comparing differences in blood type between TAVI patients and 6500 consecutive (non-TAVI) hospitalised patients, type A was significantly more prevalent in the TAVI group than in the above-mentioned control group (45 vs. 38 %, *p* = 0.023). All other blood types were distributed evenly between the TAVI and the control group.

We then divided our patients into two groups according to blood type: type A (*n* = 220) and non-type A (*n* = 271). Baseline demographic, clinical, and procedural characteristics for the two groups are presented in Table 1.

**Table 1:** Baseline study population characteristics

Units	Blood type A (*n* = 220)	Non-type A (*n* = 271)	*p* value
Age, years (mean ± SD)years	83 ± 6	83 ± 6	0.748
Male gender	86, 40%	127,47%	0.084
Diabetes mellitus	65, 29%	110, 40%	**0.011**
Dyslipidaemia	172, 78%	221, 81%	0.353
Hypertension	191, 87%	238, 88%	0.739
Smoking history	50, 23%	86, 32%	**0.027**
BMI (mean ± SD)kg/m^2^	27 ± 5	28 ± 5	0.110
CCT (mean ± SD)ml/min	63 ± 18	62 ± 20	0.770
PVD	10, 4.5%	23, 8.5%	0.083
Stroke	21, 9.5%	30, 11.1%	0.582
Systolic heart failure	36, 16%	47,17%	0.773
History of CAD	135, 61%	163, 60%	0.784
Prior MI	32, 14.5%	56, 21%	0.079
Prior CABG	39, 19%	53, 20%	0.605
Permanent pacemaker	16, 7.3%	40, 14.8%	**0.009**
AF (any type)	59, 27 %	88, 32%	0.174
EF % (mean ± SD)	56 ± 7.3	55 ± 7.7	0.253
STS score (mean ± SD)	4.2 ± 2.5	4.3 ± 3	0.510
Euroscore (mean ± SD)	25 ± 14	23 ± 14	0.259
AVA (mean ± SD)cm^2^	0.721 ± 0.19	0.714 ± 0.18	0.685
Dialysis	4, 2 %	6, 2 %	0.757
Frailty	36, 17 %	33, 12 %	0.164
PCI pre TAVI	117	141	0.800
*Medications post TAVI*			
ASA	193	226	0.178
ADP	179	226	0.557
AC	45	58	0.798
ASA + ADP	164	188	0.207
(ASA or ADP) + AC	20	29	0.555
ASA + ADP + AC	23	24	0.550
Only AC	2	5	0.385

*AC* anticoagulation, *ADP* adenosine diphosphate inhibitors, *AF* atrial fibrillation, *ASA* aspirin, *AVA* aortic valve area, *BMI* body mass index, *CABG* coronary artery bypass grafting, *CAD* coronary artery disease, *CAF* chronic atrial fibrillation, *CCT* creatinine clearance test, *COPD* chronic obstructive pulmonary disease, *EF* ejection fraction, *MI* myocardial infarction, *PCI* percutaneous coronary intervention, *PVD* peripheral vascular disease, *SD* standard deviation, *STS* Society of Thoracic Surgeons risk model, *TAVI* transcatheter aortic valve implantation

The 30-day adverse events in the type A vs. the non-type A groups are presented in Table 2. Major and fatal bleeding were more prevalent in the type A group (14.5 vs. 8.2 %, *p* = 0.027). The rate of vascular complications was higher in the type A group including both major (12.3 vs. 7 %, *p* = 0.047), as well as major or minor vascular complications (24.5 vs. 15.9 %, *p* = 0.016). There was no difference in 30-day, 1-year, and all-time mortality.

**Table 2:** Thirty-day adverse events

Events/Patient numbers	Blood type A (*n* = 220)	Non-type A (*n* = 271)	*p* value
MI	0	0	NA
Cardiogenic shock	4 (1.8 %)	6 (2.2 %)	0.757
Respiratory failure	12 (5.5 %)	17 (6.3 %)	0.702
Ventricular tachycardia	0	0	NA
Ventricular fibrillation	1 (0.5 %)	0	0.267
New atrial fibrillation	13 (5.9 %)	17 (6.3 %)	0.867
Conduction defect	73 (33.2 %)	97 (35.8 %)	0.545
Stroke	2 (0.9 %)	5 (1.8 %)	0.384
New pacemaker	40 (18.2 %)	60 (22.1 %)	0.279
Bleeding- major/fatal	32 (14.5 %)	22 (8.1 %)	**0.024**
AKI during hospitalisation	33 (15 %)	51 (18.8 %)	0.264
VC-minor	27 (12.3 %)	24 (8.9 %)	0.218
VC-major	27 (12.3 %)	19 (7 %)	**0.047**
VC-minor or major	54 (24.5 %)	43(15.9 %)	**0.016**
Surgery for VC	7 (3.2 %)	4 (1.5 %)	0.204
Use of packed cells (1 or more)	81 (36.8 %)	86 (31.7 %)	0.237
Sepsis	6 (2.7 %)	5 (1.8 %)	0.511
Conversion to open surgery	1 (0.5 %)	1 (0.4 %)	0.882
Unplanned CPB during TAVI	0	0	NA
Coronary obstruction	1 (0.5 %)	1 (0.4 %)	0.882
Ventricular septal perforation	0	0	NA
Mitral valve damage	1 (0.5 %)	1 (0.4 %)	0.882
Tamponade	3 (1.4 %)	1 (0.4 %)	0.223
Endocarditis	0	0	NA
Valve thrombosis	0	0	NA
Valve migration	0	2 (0.7 %)	0.202
Valve embolisation	1 (0.5 %)	5 (1.8 %)	0.163
TAVI-in-TAVI	0	4 (1.5 %)	0.07
30 day mortality	8 (3.6 %)	6 (2.2 %)	0.346

*AKI* acute kidney injury, *CPB* cardiopulmonary bypass, *MI* myocardial infarction, *TAVI* transcatheter aortic valve implantation, *VC* vascular complication

In multivariable linear regression analysis, blood type A was significantly and independently associated with vascular complications (OR 1.64, 95 % CI 1.04–2.67, *p* = 0.033) and was marginally associated with vascular complications or major bleeding (OR 1.57, 95 % CI 0.99–2.2167, *p* = 0.056) (Table 3).

**Table 3:** Logistic regression models

Correlates:	Model 1^a^			Model 2^b^		
	*p*	OR	95 % CI	*p*	OR	95 % CI
Age	0.701	0.991	0.946–1.038	0.849	0.996	0.951–1.042
Gender	0.192	1.397	0.845–2.311	0.141	1.476	0.879–2.479
DM	0.058	0.597	0.350–1.018	**0.017**	0.521	0.305–0.890
Hypertension	0.198	1.694	0.760–3.777	0.510	1.287	0.607–2.726
eGFR^c^	0.830	0.999	0.985–1.012	0.331	1.007	0.993–1.021
STS score	0.183	1.070	0.968–1.183	**0.036**	1.117	1.008–1.239
Prior stroke	0.227	1.560	0.759–3.205	0.342	1.421	0.688–2.934
LVEF %	0.351	1.016	0.982–1.052	0.156	1.026	0.990–1.062
Blood type A	**0.033**	1.670	1.043–2.674	**0.056**	1.576	0.989–2.510
Anaemia	0.702	1.103	0.666–1.827	0.821	0.926	0.477–1.799

*CI* confidence interval, *DM* diabetes mellitus, *LVEF* left ventricular ejection fraction, *eGFR* estimated glomerular filtration rate, *OR* odds ratio, *STS* Society of Thoracic Surgeons risk model

^a^Model 1: dependant variable: Any vascular complication

^b^Model 2: dependant variable: Any vascular complication or major bleeding

^c^eGFR in ml/min/1.73 m^2^

## Discussion

In the present study we demonstrated that among patients undergoing TAVI, blood type A was significantly more prevalent as compared with the general population, and that type A was independently and significantly associated with vascular complications.

Since 1901, when Landsteiner identified the ABO blood type system and its importance in transfusion and transplantation medicine, there has been debate regarding its usefulness as a predictor of diseases, in particular cardiovascular diseases. Carpeggiani et al. showed that non-O blood types are a predictor of increased mortality in patients with ischaemic heart disease and that it increases the risk of cardiac death amongst non-elderly patients [21]. In 1969, Jick et al. reported a deficit of patients with blood type O among those who received anticoagulants for venous thromboembolism [22]. A number of later studies elucidated that ABO blood types, particularly non-O blood types, are associated with major cardiovascular risk factors and/or increased rate of cardiovascular events [23, 24]. However, there is limited consensus regarding the magnitude and significance of the ABO effects at the population level and whether it relates to all disorders equally or predominantly modulates thrombotic pathways and disorders [25]. In addition, a genome-wide study showed an association between ABO blood types and myocardial infarction in the presence of coronary artery atherosclerosis [9]. Other reports, however, found no difference in the incidence of ABO blood type when patients with congenital and rheumatic valvular heart disease were compared with a control group [26]. Recently, various studies pointed to the association between blood type A and vascular diseases. One of them, a meta-analysis published by He et al., on 90,000 participants and more than 2 million person-years, showed that non-O blood types had a higher risk of coronary heart disease compared with blood type O [27].

Several mechanisms have been proposed to explain the possible relationship between blood types and vascular complications. ABO antigens are known to be carried by several platelet glycoproteins (GPs), for example, GPIb, GPIIb, GPIIIa, and platelet endothelial cell adhesion molecule (PECAM) [28], which play important roles in platelet function. GPIIb is an integral component of the GPIIb-GPIIIa fibrinogen receptor complex, which represents the critical final common pathway for platelet-driven thrombosis in homeostasis and pathological arterial thrombosis including acute myocardial infarction. Genetic variation in GPIIb that modulates fibrinogen binding has been associated with altered risk of thrombosis and myocardial infarction [29], so it is conceivable that ABO-driven carbohydrate modification of GPIIb might alter its functional interactions with fibrinogen and thus platelet-mediated thrombosis. However, this hypothesis has not been adequately addressed to date. Besides GPIIb and PECAM, blood type A antigen is also expressed on other uncharacterised platelet proteins. Thus these and other uncharacterised ABO-expressing platelet proteins may also act as potential functional modulators of the ABO associations with arterial thrombosis and cardiovascular events.

Previous data demonstrated that relative to non-type O, carriers of type O have significantly lower circulating plasma Von Willebrand factor (VWF) and factor VIII (FVIII) levels [30]. Although this clinically important effect of ABO type on plasma VWF-FVIII levels is well established, the mechanism through which it is mediated is not completely resolved. ABO appears to have direct functional effects on circulating VWF and indirectly (via influence of VWF levels) modulates FVIII levels. Whether VWF in platelets (a relatively abundant source) undergoes any modification by ABO remains controversial; such modification could alter platelet production and subsequent turnover of VWF, particularly locally during platelet-driven arterial thrombosis, although this remains to be established.

The limitation of this study is its relatively medium-sized study sample, a one-centre experience and the retrospective nature of the analyses. Further studies will elucidate the important questions as to whether patients with mild aortic stenosis and blood type A progress more rapidly to severe symptomatic aortic stenosis and the need for intervention and thus for a more rigorous follow-up, and whether patients with blood type A undergoing TAVI need a longer and more careful follow-up for vascular complications.

In conclusion, in this study we showed for the first time that patients referred for TAVI due to severe aortic stenosis have a higher prevalence of blood type A. We also showed that blood type A confers a higher risk of vascular complications, independent of other confounders. Future larger and multi-centre studies will assess whether blood type A is related to other aspects of diagnosis and prognosis in cardiovascular diseases and procedures.
